# 4-(2*H*-1,3-Benzodioxol-5-yl)-1-(4-methyl­phenyl)-1*H*-pyrazol-5-amine

**DOI:** 10.1107/S1600536813009914

**Published:** 2013-04-17

**Authors:** Nilesh N. Gajera, Mukesh C. Patel, Mukesh M. Jotani, Edward R. T. Tiekink

**Affiliations:** a P.S. Science and H.D. Patel Arts College, S.V. Campus, Kadi, Gujarat 382 715, India; b Department of Physics, Bhavan’s Sheth R.A. College of Science, Ahmedabad, Gujarat 380 001, India; c Department of Chemistry, University of Malaya, 50603 Kuala Lumpur, Malaysia

## Abstract

In the title compound, C_17_H_15_N_3_O_2_, two independent mol­ecules (*A* and *B)* comprise the asymmetric unit. The major conformational difference arises in the relative orientation of the pyrazole ring amine and dioxole substituents which are *anti* in *A* and *syn* in *B*. The five-membered dioxole ring in each mol­ecule has an envelope conformation with the methyl­ene C atom as the flap. The mean plane through the benzodioxole and benzene groups make dihedral angles of 31.67 (8) and 68.22 (9)°, respectively, with the pyrazole ring in *A*; the equivalent values for *B* are 47.18 (7) and 49.08 (9)°. In the crystal, supra­molecular zigzag chains along the *b-*axis direction arise as a result of N—H⋯N hydrogen bonding. These are consolidated into supra­molecular double chains *via* C—H⋯O and C—H⋯π inter­actions.

## Related literature

For background to the biological activity of amino substituted pyrazole derivatives, see: Tanitame *et al.* (2004 ▶); Chimenti *et al.* (2006 ▶); Ding *et al.* (2009 ▶); Shen *et al.* (2011 ▶); Deng *et al.* (2012 ▶). For a related structure, see: Muruganantham *et al.* (2007 ▶).
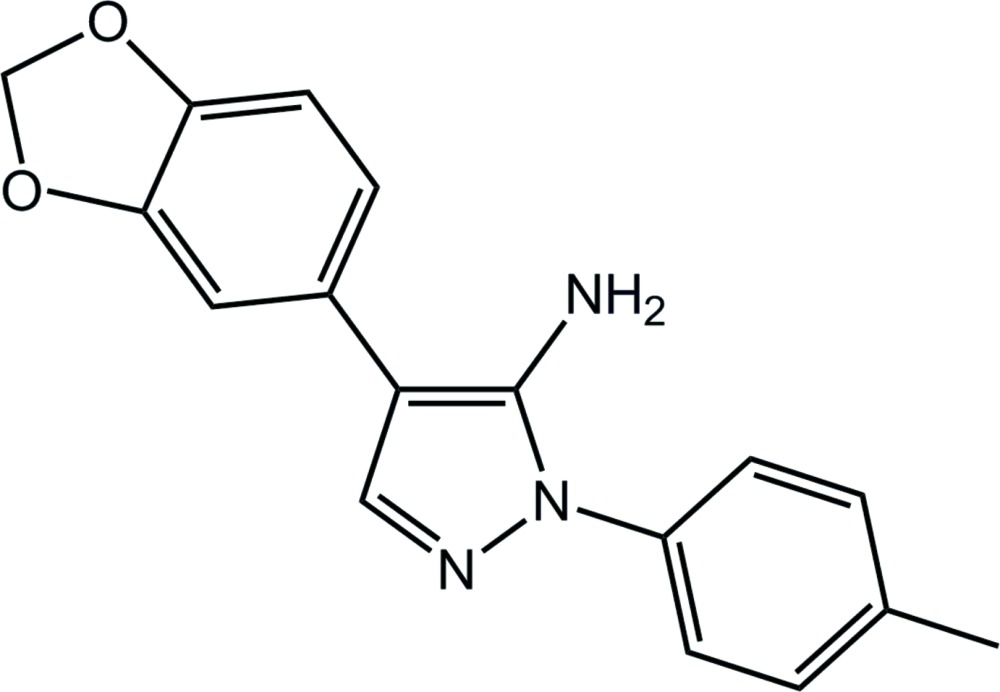


## Experimental

### 

#### Crystal data

C_17_H_15_N_3_O_2_
*M* *_r_* = 293.32
Triclinic, 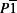

*a* = 9.7690 (7) Å
*b* = 10.4250 (7) Å
*c* = 14.283 (1) Å
α = 96.626 (2)°
β = 91.903 (2)°
γ = 91.164 (2)°
*V* = 1443.67 (17) Å^3^
*Z* = 4
Mo *K*α radiation
μ = 0.09 mm^−1^
*T* = 293 K
0.40 × 0.25 × 0.20 mm

#### Data collection

Bruker APEXII CCD diffractometer
Absorption correction: multi-scan (*SADABS*; Bruker, 2004 ▶) *T*_min_ = 0.965, *T*_max_ = 0.982
29089 measured reflections
6620 independent reflections
4674 reflections with *I* > 2σ(*I*)
*R*_int_ = 0.031

#### Refinement

*R*[*F* ^2^ > 2σ(*F* ^2^)] = 0.045
*wR*(*F* ^2^) = 0.136
*S* = 1.02
6620 reflections
412 parameters
4 restraints
H atoms treated by a mixture of independent and constrained refinement
Δρ_max_ = 0.19 e Å^−3^
Δρ_min_ = −0.17 e Å^−3^

### 

Data collection: *APEX2* (Bruker, 2004 ▶); cell refinement: *APEX2* and *SAINT* (Bruker, 2004 ▶); data reduction: *SAINT*; program(s) used to solve structure: *SHELXS97* (Sheldrick, 2008 ▶); program(s) used to refine structure: *SHELXL97* (Sheldrick, 2008 ▶); molecular graphics: *ORTEP-3 for Windows* (Farrugia, 2012 ▶), *QMol* (Gans & Shalloway, 2001 ▶) and *DIAMOND* (Brandenburg, 2006 ▶); software used to prepare material for publication: *publCIF* (Westrip, 2010 ▶).

## Supplementary Material

Crystal structure: contains datablock(s) global, I. DOI: 10.1107/S1600536813009914/su2585sup1.cif

Structure factors: contains datablock(s) I. DOI: 10.1107/S1600536813009914/su2585Isup2.hkl

Supplementary material file. DOI: 10.1107/S1600536813009914/su2585Isup3.cml

Additional supplementary materials:  crystallographic information; 3D view; checkCIF report

## Figures and Tables

**Table 1:** Hydrogen-bond geometry (Å, °) *Cg*1–*Cg*3 are the centroids of the C28–C33, C19–C24 and C2–C7 rings, respectviely.

*D*—H⋯*A*	*D*—H	H⋯*A*	*D*⋯*A*	*D*—H⋯*A*
N3—H1*N*⋯N5^i^	0.89 (2)	2.20 (2)	3.059 (2)	161 (2)
N6—H3*N*⋯N2^ii^	0.89 (1)	2.11 (1)	2.9914 (19)	170 (2)
C1—H1*C*⋯O3^ii^	0.96	2.54	3.479 (2)	164
C3—H3⋯*Cg*1^iii^	0.93	2.83	3.5365 (19)	133
C10—H10⋯*Cg*2^ii^	0.93	2.88	3.6055 (17)	135
C27—H27⋯*Cg*3^i^	0.93	2.94	3.5903 (18)	128

Symmetry codes: (i) 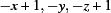; (ii) 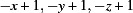; (iii) 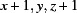.

**Fig. 1.**
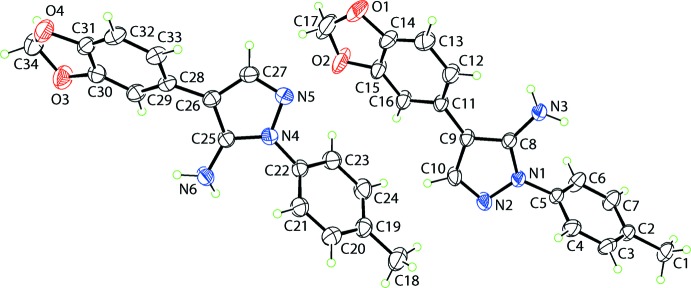
The molecular structure of the two independent molecules comprising the asymmetric unit of the title compound (I), showing the atom labelling. Displacement ellipsoids are drawn at the 35% probability level.

**Fig. 2.**
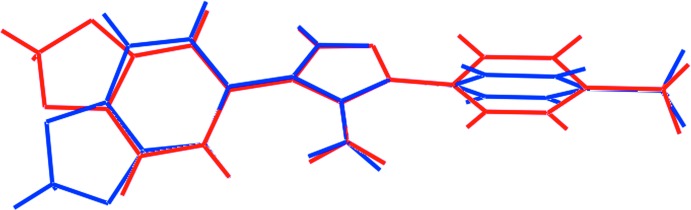
Overlay diagram of the N1- and inverted N2-containing molecules (red and blue, respectively), where the pyrazole rings have been superimposed.

**Fig. 3.**
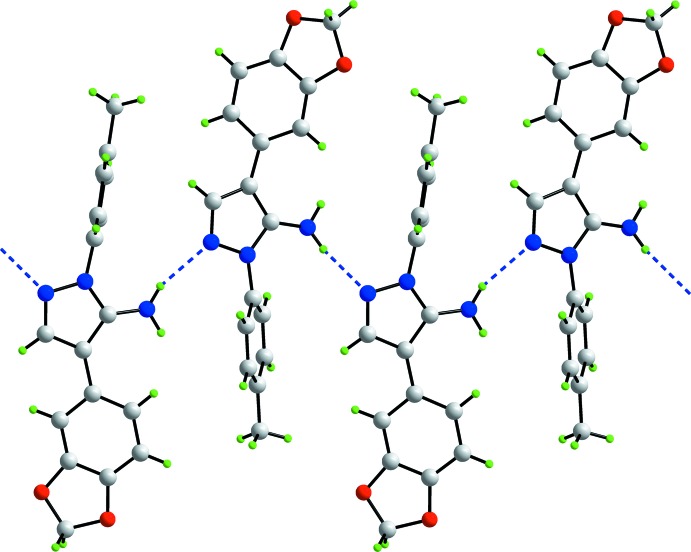
View of the supramolecular zigzag chain along the b axis in compound (I), mediated by N—H···N hydrogen bonds (dashed lines; see Table 1 for details).

**Fig. 4.**
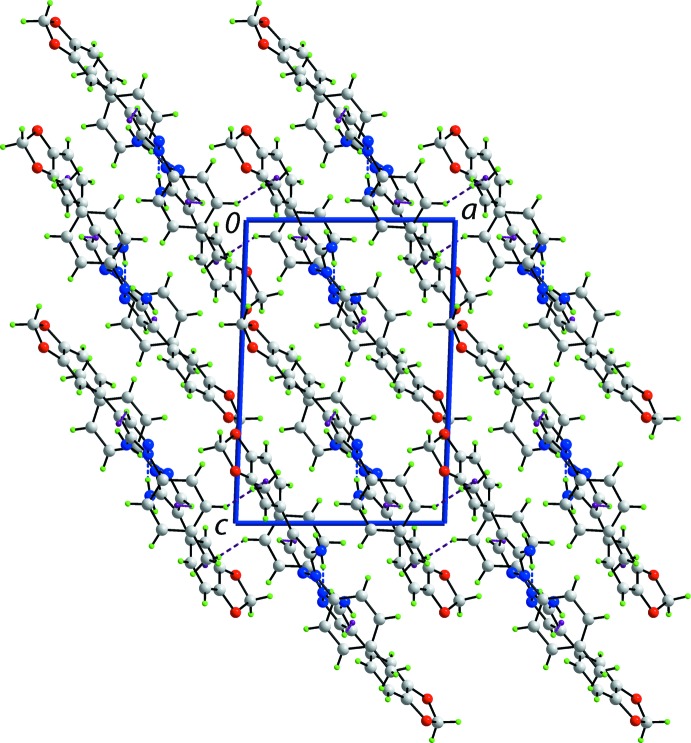
A view in projection along the b axis of the crystal packing of compound (I). The N—H···N, C—H···O (obscured) and C—H···π interactions are shown as dashed lines (see Table 1 for details).

## supplementary crystallographic information

### Comment

The amino substituted pyrazole unit is an important backbone in the area of synthetic as well as medicinal chemistry due to the broad range of biological activities of such compounds, such as anti-depressant, anti-anxiety, anti-fungal, anti-bacterial, anti-diabetic and anti-cancer (Tanitame *et al.*, 2004; Chimenti *et al.*, 2006; Ding *et al.*,2009; Shen *et al.*, 2011; Deng *et al.*, 2012). In this connection, the title compound, (I), was synthesized and its crystal structure is reported on herein.

Two independent molecules (A and B), comprise the asymmetric unit of (I), see Fig. 1. As seen from the overlay diagram, Fig. 2, different conformations are observed for both the benzodioxole and benzene substituents. The five-membered dioxole rings in each molecule has an envelope conformation with the methylene-C17 or C34 atoms being the flap atoms. The r.m.s. deviation of the five non-hydrogen atoms = 0.049 Å for the N1-containing molecule which is considerably less than 0.129 Å for the second independent molecule, where the C34 atom lies 0.115 (2) Å out of the mean plane. For the N1-containing molecule, with respect to the pyrazole ring (r.m.s. deviation = Å), the benzodioxole and benzene groups make dihedral angles of 31.67 (8) and 68.22 (9)°, respectively. The equivalent values for the N2-containing molecule are 47.18 (7)° and 49.08 (9)°, respectively. From a conformational point of view, the dioxole ring in the N1-containing molecule is *anti* to the amine substituent whereas it is *syn* for the second molecule. The observed conformations in (I) are similar to those in a closely related structure, *i.e.* diethyl 4-(benzo[*d*][1,3]dioxol-5-yl)-1*H*-pyrazol-3-yl-3-phosphonate (Muruganantham *et al.*, 2007).

The presence of N—H···N hydrogen bonding leads to supramolecular zigzag chains along the *b* axis in the crystal structure of (I), see Fig. 3 and Table 1. These are consolidated into supramolecular double chains *via* C—H···O and C—H···π interactions (Table 1). These stack with no specific intermolecular interactions between them (Fig. 4).

### Experimental

A mixture of 3,4-methyleneoxyphenyl acetonitrile (2 g, 0.012 mol) and *N*,*N*-dimethylformamide dimethylacetal (4.89 ml, 0.037 mol) was stirred at 355 K; progress of the reaction was monitored by TLC. At the end of the reaction, the solvent was removed under vacuum. The residual crude mass was mixed with 4-methyl phenyl hydrazine hydrochloride (1.96 g, 0.012 mol) in methanol (20 ml) at room temperature. The mixture was refluxed and the reaction progress was monitored by TLC. At the end of the reaction, the solvent was removed under reduced pressure. The residue was dissolved in water and NaHCO_3_ solution was added until basic pH was obtained. The product was extracted in ethyl acetate (200 ml × 2), and this ethyl acetate layer passed through Na_2_SO_4_ and concentrated to dryness. The crude mass was purified by silica gel column chromatography, eluted with ethyl acetate:hexane (1:4) to produced 2.8 g of a yellow solid [Yield: 77%. *M*.pt: 422–423 K]. Single crystals suitable for X-ray measurements were obtained by repeated re-crystallization from ethyl acetate at room temperature.

### Refinement

The C-bound H atoms were geometrically placed (C—H = 0.93–0.97 Å) and refined as riding with *U_iso_*(H) = 1.2–1.5*U_eq_*(C). The N-bound H-atom was refined with the distance restraint N—H = 0.89 (1) Å, and with *U_iso_*(H) = 1.2*U_eq_*(N). Being affected by the beam-stop, the (0 0 1) reflection was removed from the final cycles of refinement.

### Crystal data

**Table:**

C_17_H_15_N_3_O_2_	*Z* = 4
*M**_r_* = 293.32	*F*(000) = 616
Triclinic, *P*1	*D*_x_ = 1.350 Mg m^−^^3^
Hall symbol: -P 1	Mo *K*α radiation, λ = 0.71069 Å
*a* = 9.7690 (7) Å	Cell parameters from 5105 reflections
*b* = 10.4250 (7) Å	θ = 2.5–28.1°
*c* = 14.283 (1) Å	µ = 0.09 mm^−^^1^
α = 96.626 (2)°	*T* = 293 K
β = 91.903 (2)°	Block, light-brown
γ = 91.164 (2)°	0.40 × 0.25 × 0.20 mm
*V* = 1443.67 (17) Å^3^	

### Data collection

**Table:**

Bruker APEXII CCD diffractometer	6620 independent reflections
Radiation source: fine-focus sealed tube	4674 reflections with *I* > 2σ(*I*)
Graphite monochromator	*R*_int_ = 0.031
Detector resolution: 10.0 pixels mm^-1^	θ_max_ = 27.5°, θ_min_ = 2.0°
ω and φ scan	*h* = −12→12
Absorption correction: multi-scan (*SADABS*; Bruker, 2004)	*k* = −13→13
*T*_min_ = 0.965, *T*_max_ = 0.982	*l* = −17→18
29089 measured reflections	

### Refinement

**Table:**

Refinement on *F*^2^	Secondary atom site location: difference Fourier map
Least-squares matrix: full	Hydrogen site location: inferred from neighbouring sites
*R*[*F*^2^ > 2σ(*F*^2^)] = 0.045	H atoms treated by a mixture of independent and constrained refinement
*wR*(*F*^2^) = 0.136	*w* = 1/[σ^2^(*F*_o_^2^) + (0.0657*P*)^2^ + 0.2775*P*] where *P* = (*F*_o_^2^ + 2*F*_c_^2^)/3
*S* = 1.02	(Δ/σ)_max_ = 0.001
6620 reflections	Δρ_max_ = 0.19 e Å^−^^3^
412 parameters	Δρ_min_ = −0.17 e Å^−^^3^
4 restraints	Extinction correction: *SHELXL97* (Sheldrick, 2008), Fc^*^=kFc[1+0.001xFc^2^λ^3^/sin(2θ)]^-1/4^
Primary atom site location: structure-invariant direct methods	Extinction coefficient: 0.0100 (15)

### Special details

**Table:**

Geometry. All s.u.'s (except the s.u. in the dihedral angle between two l.s. planes) are estimated using the full covariance matrix. The cell s.u.'s are taken into account individually in the estimation of s.u.'s in distances, angles and torsion angles; correlations between s.u.'s in cell parameters are only used when they are defined by crystal symmetry. An approximate (isotropic) treatment of cell s.u.'s is used for estimating s.u.'s involving l.s. planes.
Refinement. Refinement of *F*^2^ against ALL reflections. The weighted *R*-factor *wR* and goodness of fit *S* are based on *F*^2^, conventional *R*-factors *R* are based on *F*, with *F* set to zero for negative *F*^2^. The threshold expression of *F*^2^ > 2σ(*F*^2^) is used only for calculating *R*-factors(gt) *etc*. and is not relevant to the choice of reflections for refinement. *R*-factors based on *F*^2^ are statistically about twice as large as those based on *F*, and *R*- factors based on ALL data will be even larger.

### Fractional atomic coordinates and isotropic or equivalent isotropic displacement parameters (Å^2^)

**Table:**

	*x*	*y*	*z*	*U*_iso_*/*U*_eq_	
O1	0.05184 (17)	0.01597 (17)	0.34864 (10)	0.0926 (5)	
O2	0.05160 (17)	0.22486 (16)	0.42087 (11)	0.0941 (5)	
N1	0.56918 (13)	0.17929 (11)	0.77181 (8)	0.0456 (3)	
N2	0.56849 (16)	0.29926 (12)	0.74072 (9)	0.0558 (4)	
N3	0.46446 (17)	−0.02875 (14)	0.74119 (11)	0.0629 (4)	
H1N	0.5319 (15)	−0.0597 (18)	0.7748 (12)	0.075*	
H2N	0.4200 (19)	−0.0813 (16)	0.6962 (11)	0.075*	
C1	0.8680 (2)	0.1459 (2)	1.11796 (13)	0.0687 (5)	
H1A	0.9252	0.0718	1.1123	0.103*	
H1B	0.8048	0.1390	1.1673	0.103*	
H1C	0.9241	0.2228	1.1328	0.103*	
C2	0.78997 (16)	0.15254 (14)	1.02637 (11)	0.0478 (4)	
C3	0.85553 (17)	0.16857 (19)	0.94457 (13)	0.0624 (5)	
H3	0.9506	0.1765	0.9464	0.075*	
C4	0.78454 (17)	0.17326 (18)	0.85982 (12)	0.0583 (4)	
H4	0.8314	0.1825	0.8053	0.070*	
C5	0.64441 (15)	0.16413 (13)	0.85703 (10)	0.0406 (3)	
C6	0.57641 (16)	0.14683 (18)	0.93748 (11)	0.0564 (4)	
H6	0.4813	0.1391	0.9355	0.068*	
C7	0.64917 (17)	0.14102 (18)	1.02109 (11)	0.0575 (4)	
H7	0.6022	0.1291	1.0751	0.069*	
C8	0.48562 (15)	0.09408 (13)	0.71784 (10)	0.0415 (3)	
C9	0.42651 (15)	0.15896 (13)	0.64773 (9)	0.0404 (3)	
C10	0.48195 (18)	0.28428 (15)	0.66748 (10)	0.0513 (4)	
H10	0.4597	0.3509	0.6321	0.062*	
C11	0.32854 (15)	0.11204 (14)	0.57068 (10)	0.0420 (3)	
C12	0.32865 (19)	−0.01296 (16)	0.52498 (12)	0.0569 (4)	
H12	0.3908	−0.0707	0.5458	0.068*	
C13	0.2395 (2)	−0.05524 (18)	0.44922 (13)	0.0680 (5)	
H13	0.2408	−0.1394	0.4195	0.082*	
C14	0.15023 (19)	0.03216 (19)	0.42066 (12)	0.0610 (5)	
C15	0.14910 (18)	0.15617 (18)	0.46424 (12)	0.0578 (4)	
C16	0.23427 (17)	0.19885 (15)	0.53917 (11)	0.0509 (4)	
H16	0.2301	0.2830	0.5686	0.061*	
C17	−0.0015 (3)	0.1391 (3)	0.34386 (19)	0.1104 (9)	
H17A	−0.1007	0.1344	0.3455	0.132*	
H17B	0.0230	0.1703	0.2851	0.132*	
O3	−0.00982 (14)	0.54440 (13)	−0.17121 (10)	0.0746 (4)	
O4	−0.01550 (13)	0.38075 (14)	−0.29402 (9)	0.0685 (4)	
N4	0.39249 (13)	0.31673 (12)	0.17245 (9)	0.0458 (3)	
N5	0.33173 (15)	0.19573 (12)	0.16344 (10)	0.0563 (4)	
N6	0.41337 (19)	0.50194 (14)	0.09039 (10)	0.0634 (4)	
H3N	0.427 (2)	0.5557 (16)	0.1430 (9)	0.076*	
H4N	0.386 (2)	0.5369 (18)	0.0393 (10)	0.076*	
C18	0.7688 (2)	0.4236 (2)	0.48865 (14)	0.0753 (6)	
H18A	0.7271	0.3984	0.5438	0.113*	
H18B	0.7928	0.5140	0.4987	0.113*	
H18C	0.8498	0.3747	0.4765	0.113*	
C19	0.66949 (18)	0.39817 (15)	0.40553 (12)	0.0525 (4)	
C20	0.70590 (18)	0.42017 (18)	0.31628 (13)	0.0586 (4)	
H20	0.7938	0.4516	0.3077	0.070*	
C21	0.61593 (17)	0.39708 (17)	0.23905 (12)	0.0534 (4)	
H21	0.6435	0.4125	0.1796	0.064*	
C22	0.48521 (16)	0.35108 (14)	0.25076 (10)	0.0430 (3)	
C23	0.44556 (18)	0.32943 (17)	0.33975 (11)	0.0539 (4)	
H23	0.3571	0.2995	0.3486	0.065*	
C24	0.5377 (2)	0.35237 (17)	0.41535 (11)	0.0587 (4)	
H24	0.5103	0.3365	0.4748	0.070*	
C25	0.36066 (16)	0.38099 (14)	0.09701 (10)	0.0418 (3)	
C26	0.27733 (15)	0.29921 (14)	0.03560 (10)	0.0427 (3)	
C27	0.26475 (17)	0.18800 (15)	0.08121 (12)	0.0532 (4)	
H27	0.2137	0.1152	0.0558	0.064*	
C28	0.21039 (14)	0.32219 (14)	−0.05457 (10)	0.0424 (3)	
C29	0.14039 (16)	0.43657 (15)	−0.06373 (11)	0.0480 (4)	
H29	0.1429	0.5044	−0.0153	0.058*	
C30	0.06824 (16)	0.44450 (15)	−0.14665 (12)	0.0500 (4)	
C31	0.06488 (15)	0.34652 (17)	−0.21981 (11)	0.0487 (4)	
C32	0.13435 (16)	0.23582 (17)	−0.21447 (11)	0.0524 (4)	
H32	0.1332	0.1702	−0.2645	0.063*	
C33	0.20731 (15)	0.22566 (15)	−0.13039 (11)	0.0484 (4)	
H33	0.2561	0.1511	−0.1247	0.058*	
C34	−0.0848 (2)	0.4919 (2)	−0.25516 (15)	0.0751 (6)	
H34A	−0.1776	0.4686	−0.2404	0.090*	
H34B	−0.0891	0.5551	−0.2999	0.090*	

### Atomic displacement parameters (Å^2^)

**Table:**

	*U*^11^	*U*^22^	*U*^33^	*U*^12^	*U*^13^	*U*^23^
O1	0.1014 (11)	0.1036 (12)	0.0658 (9)	−0.0138 (9)	−0.0459 (8)	−0.0029 (8)
O2	0.1030 (11)	0.0994 (11)	0.0758 (10)	0.0264 (9)	−0.0522 (9)	0.0041 (8)
N1	0.0574 (8)	0.0396 (6)	0.0388 (7)	−0.0019 (5)	−0.0142 (6)	0.0058 (5)
N2	0.0851 (10)	0.0404 (7)	0.0410 (7)	−0.0072 (6)	−0.0169 (7)	0.0076 (5)
N3	0.0804 (11)	0.0434 (7)	0.0639 (10)	−0.0094 (7)	−0.0347 (8)	0.0143 (6)
C1	0.0753 (13)	0.0721 (12)	0.0569 (11)	−0.0031 (10)	−0.0314 (9)	0.0100 (9)
C2	0.0530 (9)	0.0442 (8)	0.0448 (8)	−0.0021 (7)	−0.0169 (7)	0.0045 (6)
C3	0.0398 (9)	0.0831 (12)	0.0649 (11)	−0.0149 (8)	−0.0139 (8)	0.0191 (9)
C4	0.0478 (9)	0.0790 (12)	0.0498 (9)	−0.0143 (8)	−0.0029 (7)	0.0188 (8)
C5	0.0467 (8)	0.0388 (7)	0.0352 (7)	−0.0006 (6)	−0.0108 (6)	0.0034 (6)
C6	0.0380 (8)	0.0868 (12)	0.0438 (9)	0.0081 (8)	−0.0041 (7)	0.0053 (8)
C7	0.0527 (10)	0.0838 (12)	0.0359 (8)	0.0059 (9)	−0.0010 (7)	0.0060 (8)
C8	0.0457 (8)	0.0405 (7)	0.0374 (7)	0.0016 (6)	−0.0081 (6)	0.0029 (6)
C9	0.0477 (8)	0.0417 (7)	0.0314 (7)	0.0060 (6)	−0.0039 (6)	0.0027 (6)
C10	0.0778 (11)	0.0413 (8)	0.0344 (8)	0.0026 (7)	−0.0111 (7)	0.0064 (6)
C11	0.0487 (8)	0.0456 (8)	0.0312 (7)	0.0033 (6)	−0.0052 (6)	0.0036 (6)
C12	0.0684 (11)	0.0495 (9)	0.0505 (9)	0.0083 (8)	−0.0148 (8)	−0.0011 (7)
C13	0.0876 (14)	0.0545 (10)	0.0567 (11)	−0.0033 (9)	−0.0176 (10)	−0.0094 (8)
C14	0.0654 (11)	0.0727 (12)	0.0416 (9)	−0.0104 (9)	−0.0182 (8)	0.0011 (8)
C15	0.0610 (10)	0.0688 (11)	0.0435 (9)	0.0069 (8)	−0.0157 (8)	0.0106 (8)
C16	0.0639 (10)	0.0485 (8)	0.0391 (8)	0.0091 (7)	−0.0119 (7)	0.0018 (6)
C17	0.119 (2)	0.117 (2)	0.0897 (18)	0.0012 (17)	−0.0649 (16)	0.0106 (15)
O3	0.0836 (9)	0.0649 (8)	0.0748 (9)	0.0165 (7)	−0.0190 (7)	0.0105 (6)
O4	0.0636 (8)	0.0895 (10)	0.0523 (7)	0.0004 (7)	−0.0138 (6)	0.0123 (6)
N4	0.0543 (8)	0.0406 (6)	0.0420 (7)	−0.0039 (6)	−0.0040 (6)	0.0051 (5)
N5	0.0611 (9)	0.0426 (7)	0.0653 (9)	−0.0088 (6)	−0.0129 (7)	0.0122 (6)
N6	0.1031 (12)	0.0469 (8)	0.0389 (8)	−0.0208 (8)	−0.0140 (8)	0.0076 (6)
C18	0.0871 (14)	0.0711 (12)	0.0653 (12)	0.0104 (10)	−0.0261 (11)	0.0042 (10)
C19	0.0632 (11)	0.0436 (8)	0.0494 (9)	0.0081 (7)	−0.0097 (8)	0.0023 (7)
C20	0.0499 (9)	0.0651 (11)	0.0609 (11)	−0.0029 (8)	−0.0042 (8)	0.0102 (8)
C21	0.0544 (10)	0.0629 (10)	0.0440 (9)	−0.0031 (8)	0.0029 (7)	0.0109 (7)
C22	0.0523 (9)	0.0383 (7)	0.0379 (8)	0.0025 (6)	−0.0025 (6)	0.0028 (6)
C23	0.0564 (10)	0.0600 (10)	0.0450 (9)	−0.0057 (8)	0.0047 (7)	0.0055 (7)
C24	0.0777 (12)	0.0623 (10)	0.0360 (8)	0.0017 (9)	0.0020 (8)	0.0058 (7)
C25	0.0510 (8)	0.0396 (7)	0.0340 (7)	0.0004 (6)	0.0014 (6)	0.0005 (6)
C26	0.0402 (8)	0.0420 (8)	0.0440 (8)	0.0027 (6)	−0.0014 (6)	−0.0018 (6)
C27	0.0508 (9)	0.0422 (8)	0.0648 (11)	−0.0065 (7)	−0.0122 (8)	0.0041 (7)
C28	0.0353 (7)	0.0480 (8)	0.0425 (8)	−0.0029 (6)	0.0001 (6)	0.0000 (6)
C29	0.0497 (9)	0.0464 (8)	0.0459 (8)	0.0007 (7)	−0.0007 (7)	−0.0025 (6)
C30	0.0478 (9)	0.0492 (9)	0.0535 (9)	0.0018 (7)	−0.0012 (7)	0.0088 (7)
C31	0.0394 (8)	0.0666 (10)	0.0399 (8)	−0.0067 (7)	−0.0023 (6)	0.0070 (7)
C32	0.0455 (9)	0.0631 (10)	0.0444 (9)	−0.0042 (7)	0.0002 (7)	−0.0098 (7)
C33	0.0403 (8)	0.0503 (9)	0.0517 (9)	0.0030 (6)	−0.0020 (7)	−0.0061 (7)
C34	0.0674 (12)	0.0865 (14)	0.0729 (13)	0.0056 (11)	−0.0168 (10)	0.0199 (11)

### Geometric parameters (Å, º)

**Table:**

O1—C14	1.378 (2)	O3—C30	1.3741 (19)
O1—C17	1.403 (3)	O3—C34	1.431 (2)
O2—C15	1.376 (2)	O4—C31	1.3824 (19)
O2—C17	1.413 (3)	O4—C34	1.421 (3)
N1—C8	1.3492 (18)	N4—C25	1.3635 (18)
N1—N2	1.3746 (17)	N4—N5	1.3737 (18)
N1—C5	1.4270 (17)	N4—C22	1.4237 (19)
N2—C10	1.3159 (19)	N5—C27	1.319 (2)
N3—C8	1.373 (2)	N6—C25	1.367 (2)
N3—H1N	0.890 (9)	N6—H3N	0.889 (9)
N3—H2N	0.891 (9)	N6—H4N	0.888 (9)
C1—C2	1.501 (2)	C18—C19	1.504 (2)
C1—H1A	0.9600	C18—H18A	0.9600
C1—H1B	0.9600	C18—H18B	0.9600
C1—H1C	0.9600	C18—H18C	0.9600
C2—C3	1.376 (2)	C19—C20	1.378 (2)
C2—C7	1.377 (2)	C19—C24	1.381 (3)
C3—C4	1.381 (2)	C20—C21	1.383 (2)
C3—H3	0.9300	C20—H20	0.9300
C4—C5	1.369 (2)	C21—C22	1.377 (2)
C4—H4	0.9300	C21—H21	0.9300
C5—C6	1.374 (2)	C22—C23	1.383 (2)
C6—C7	1.377 (2)	C23—C24	1.379 (2)
C6—H6	0.9300	C23—H23	0.9300
C7—H7	0.9300	C24—H24	0.9300
C8—C9	1.3884 (19)	C25—C26	1.382 (2)
C9—C10	1.398 (2)	C26—C27	1.399 (2)
C9—C11	1.4674 (19)	C26—C28	1.472 (2)
C10—H10	0.9300	C27—H27	0.9300
C11—C12	1.389 (2)	C28—C33	1.390 (2)
C11—C16	1.404 (2)	C28—C29	1.403 (2)
C12—C13	1.391 (2)	C29—C30	1.370 (2)
C12—H12	0.9300	C29—H29	0.9300
C13—C14	1.361 (3)	C30—C31	1.374 (2)
C13—H13	0.9300	C31—C32	1.359 (2)
C14—C15	1.369 (3)	C32—C33	1.392 (2)
C15—C16	1.361 (2)	C32—H32	0.9300
C16—H16	0.9300	C33—H33	0.9300
C17—H17A	0.9700	C34—H34A	0.9700
C17—H17B	0.9700	C34—H34B	0.9700
			
C14—O1—C17	105.27 (16)	C30—O3—C34	104.72 (14)
C15—O2—C17	105.11 (17)	C31—O4—C34	104.44 (13)
C8—N1—N2	111.81 (12)	C25—N4—N5	111.66 (12)
C8—N1—C5	129.50 (12)	C25—N4—C22	130.28 (12)
N2—N1—C5	118.45 (12)	N5—N4—C22	117.78 (12)
C10—N2—N1	103.64 (12)	C27—N5—N4	103.58 (12)
C8—N3—H1N	115.8 (13)	C25—N6—H3N	118.6 (13)
C8—N3—H2N	113.6 (13)	C25—N6—H4N	115.1 (13)
H1N—N3—H2N	119.2 (19)	H3N—N6—H4N	116.5 (19)
C2—C1—H1A	109.5	C19—C18—H18A	109.5
C2—C1—H1B	109.5	C19—C18—H18B	109.5
H1A—C1—H1B	109.5	H18A—C18—H18B	109.5
C2—C1—H1C	109.5	C19—C18—H18C	109.5
H1A—C1—H1C	109.5	H18A—C18—H18C	109.5
H1B—C1—H1C	109.5	H18B—C18—H18C	109.5
C3—C2—C7	117.43 (14)	C20—C19—C24	117.10 (15)
C3—C2—C1	121.71 (15)	C20—C19—C18	121.41 (17)
C7—C2—C1	120.86 (16)	C24—C19—C18	121.49 (16)
C2—C3—C4	122.06 (15)	C19—C20—C21	122.14 (17)
C2—C3—H3	119.0	C19—C20—H20	118.9
C4—C3—H3	119.0	C21—C20—H20	118.9
C5—C4—C3	119.24 (16)	C22—C21—C20	119.58 (15)
C5—C4—H4	120.4	C22—C21—H21	120.2
C3—C4—H4	120.4	C20—C21—H21	120.2
C4—C5—C6	119.89 (14)	C21—C22—C23	119.46 (15)
C4—C5—N1	119.88 (14)	C21—C22—N4	121.55 (13)
C6—C5—N1	120.15 (13)	C23—C22—N4	118.85 (14)
C5—C6—C7	119.95 (15)	C24—C23—C22	119.72 (16)
C5—C6—H6	120.0	C24—C23—H23	120.1
C7—C6—H6	120.0	C22—C23—H23	120.1
C2—C7—C6	121.39 (15)	C23—C24—C19	121.99 (15)
C2—C7—H7	119.3	C23—C24—H24	119.0
C6—C7—H7	119.3	C19—C24—H24	119.0
N1—C8—N3	120.78 (13)	N4—C25—N6	121.33 (13)
N1—C8—C9	107.35 (12)	N4—C25—C26	107.16 (13)
N3—C8—C9	131.66 (14)	N6—C25—C26	131.45 (14)
C8—C9—C10	103.36 (12)	C25—C26—C27	103.66 (13)
C8—C9—C11	129.93 (13)	C25—C26—C28	129.50 (14)
C10—C9—C11	126.70 (13)	C27—C26—C28	126.78 (14)
N2—C10—C9	113.82 (13)	N5—C27—C26	113.94 (14)
N2—C10—H10	123.1	N5—C27—H27	123.0
C9—C10—H10	123.1	C26—C27—H27	123.0
C12—C11—C16	118.51 (14)	C33—C28—C29	118.87 (14)
C12—C11—C9	122.89 (13)	C33—C28—C26	119.78 (13)
C16—C11—C9	118.54 (13)	C29—C28—C26	121.19 (13)
C11—C12—C13	122.64 (15)	C30—C29—C28	117.52 (14)
C11—C12—H12	118.7	C30—C29—H29	121.2
C13—C12—H12	118.7	C28—C29—H29	121.2
C14—C13—C12	116.94 (16)	C29—C30—C31	122.34 (15)
C14—C13—H13	121.5	C29—C30—O3	128.16 (15)
C12—C13—H13	121.5	C31—C30—O3	109.50 (14)
C13—C14—C15	121.39 (15)	C32—C31—C30	121.80 (15)
C13—C14—O1	128.86 (17)	C32—C31—O4	128.42 (15)
C15—C14—O1	109.75 (16)	C30—C31—O4	109.78 (15)
C16—C15—C14	122.54 (15)	C31—C32—C33	116.63 (14)
C16—C15—O2	127.73 (17)	C31—C32—H32	121.7
C14—C15—O2	109.74 (15)	C33—C32—H32	121.7
C15—C16—C11	117.97 (15)	C28—C33—C32	122.80 (15)
C15—C16—H16	121.0	C28—C33—H33	118.6
C11—C16—H16	121.0	C32—C33—H33	118.6
O1—C17—O2	109.53 (17)	O4—C34—O3	107.51 (14)
O1—C17—H17A	109.8	O4—C34—H34A	110.2
O2—C17—H17A	109.8	O3—C34—H34A	110.2
O1—C17—H17B	109.8	O4—C34—H34B	110.2
O2—C17—H17B	109.8	O3—C34—H34B	110.2
H17A—C17—H17B	108.2	H34A—C34—H34B	108.5
			
C8—N1—N2—C10	0.48 (18)	C25—N4—N5—C27	0.77 (18)
C5—N1—N2—C10	−174.38 (13)	C22—N4—N5—C27	−173.75 (13)
C7—C2—C3—C4	0.1 (3)	C24—C19—C20—C21	−0.4 (3)
C1—C2—C3—C4	179.25 (17)	C18—C19—C20—C21	179.89 (16)
C2—C3—C4—C5	1.2 (3)	C19—C20—C21—C22	0.3 (3)
C3—C4—C5—C6	−1.8 (3)	C20—C21—C22—C23	0.4 (2)
C3—C4—C5—N1	174.93 (15)	C20—C21—C22—N4	−175.21 (14)
C8—N1—C5—C4	117.59 (19)	C25—N4—C22—C21	−46.4 (2)
N2—N1—C5—C4	−68.6 (2)	N5—N4—C22—C21	126.91 (16)
C8—N1—C5—C6	−65.7 (2)	C25—N4—C22—C23	137.94 (17)
N2—N1—C5—C6	108.15 (18)	N5—N4—C22—C23	−48.74 (19)
C4—C5—C6—C7	1.2 (3)	C21—C22—C23—C24	−0.9 (2)
N1—C5—C6—C7	−175.60 (15)	N4—C22—C23—C24	174.84 (14)
C3—C2—C7—C6	−0.8 (3)	C22—C23—C24—C19	0.7 (3)
C1—C2—C7—C6	−179.96 (17)	C20—C19—C24—C23	−0.1 (3)
C5—C6—C7—C2	0.2 (3)	C18—C19—C24—C23	179.61 (16)
N2—N1—C8—N3	−175.36 (15)	N5—N4—C25—N6	−178.24 (15)
C5—N1—C8—N3	−1.2 (2)	C22—N4—C25—N6	−4.6 (2)
N2—N1—C8—C9	0.06 (18)	N5—N4—C25—C26	−0.68 (17)
C5—N1—C8—C9	174.19 (14)	C22—N4—C25—C26	172.97 (14)
N1—C8—C9—C10	−0.53 (17)	N4—C25—C26—C27	0.29 (16)
N3—C8—C9—C10	174.19 (18)	N6—C25—C26—C27	177.51 (18)
N1—C8—C9—C11	179.89 (14)	N4—C25—C26—C28	177.61 (14)
N3—C8—C9—C11	−5.4 (3)	N6—C25—C26—C28	−5.2 (3)
N1—N2—C10—C9	−0.85 (19)	N4—N5—C27—C26	−0.59 (19)
C8—C9—C10—N2	0.89 (19)	C25—C26—C27—N5	0.20 (19)
C11—C9—C10—N2	−179.51 (14)	C28—C26—C27—N5	−177.22 (14)
C8—C9—C11—C12	−34.1 (2)	C25—C26—C28—C33	138.92 (16)
C10—C9—C11—C12	146.40 (18)	C27—C26—C28—C33	−44.3 (2)
C8—C9—C11—C16	148.63 (16)	C25—C26—C28—C29	−45.8 (2)
C10—C9—C11—C16	−30.9 (2)	C27—C26—C28—C29	130.89 (17)
C16—C11—C12—C13	0.2 (3)	C33—C28—C29—C30	2.3 (2)
C9—C11—C12—C13	−177.07 (17)	C26—C28—C29—C30	−172.96 (14)
C11—C12—C13—C14	0.1 (3)	C28—C29—C30—C31	−1.2 (2)
C12—C13—C14—C15	0.4 (3)	C28—C29—C30—O3	178.57 (16)
C12—C13—C14—O1	−179.75 (19)	C34—O3—C30—C29	−167.77 (18)
C17—O1—C14—C13	−174.4 (2)	C34—O3—C30—C31	11.99 (19)
C17—O1—C14—C15	5.4 (2)	C29—C30—C31—C32	−0.6 (3)
C13—C14—C15—C16	−1.3 (3)	O3—C30—C31—C32	179.63 (15)
O1—C14—C15—C16	178.84 (17)	C29—C30—C31—O4	179.92 (15)
C13—C14—C15—O2	178.86 (19)	O3—C30—C31—O4	0.15 (19)
O1—C14—C15—O2	−1.0 (2)	C34—O4—C31—C32	168.27 (18)
C17—O2—C15—C16	176.4 (2)	C34—O4—C31—C30	−12.29 (19)
C17—O2—C15—C14	−3.8 (3)	C30—C31—C32—C33	1.1 (2)
C14—C15—C16—C11	1.6 (3)	O4—C31—C32—C33	−179.54 (15)
O2—C15—C16—C11	−178.62 (18)	C29—C28—C33—C32	−1.9 (2)
C12—C11—C16—C15	−1.0 (2)	C26—C28—C33—C32	173.45 (14)
C9—C11—C16—C15	176.39 (15)	C31—C32—C33—C28	0.2 (2)
C14—O1—C17—O2	−7.8 (3)	C31—O4—C34—O3	19.6 (2)
C15—O2—C17—O1	7.2 (3)	C30—O3—C34—O4	−19.6 (2)

### Hydrogen-bond geometry (Å, º)

Cg1–Cg3 are the centroids of the C28–C33, C19–C24 and C2–C7 rings, respectviely.

**Table:**

*D*—H···*A*	*D*—H	H···*A*	*D*···*A*	*D*—H···*A*
N3—H1*N*···N5^i^	0.89 (2)	2.20 (2)	3.059 (2)	161 (2)
N6—H3*N*···N2^ii^	0.89 (1)	2.11 (1)	2.9914 (19)	170 (2)
C1—H1*C*···O3^ii^	0.96	2.54	3.479 (2)	164
C3—H3···*Cg*1^iii^	0.93	2.83	3.5365 (19)	133
C10—H10···*Cg*2^ii^	0.93	2.88	3.6055 (17)	135
C27—H27···*Cg*3^i^	0.93	2.94	3.5903 (18)	128

Symmetry codes: (i) −*x*+1, −*y*, −*z*+1; (ii) −*x*+1, −*y*+1, −*z*+1; (iii) *x*+1, *y*, *z*+1.
